# Advances, applications, and limitations of portable and rapid detection technologies for routinely encountered foodborne pathogens

**DOI:** 10.3389/fmicb.2022.1054782

**Published:** 2022-12-05

**Authors:** Irwin A. Quintela, Tyler Vasse, Chih-Sheng Lin, Vivian C. H. Wu

**Affiliations:** ^1^Produce Safety and Microbiology Research Unit, U.S. Department of Agriculture, Agricultural Research Service, Western Regional Research Center, Albany, CA, United States; ^2^Department of Biological Science and Technology, National Yang Ming Chiao Tung University, Hsinchu, Taiwan; ^3^Department of Biological Science and Technology, National Chiao Tung University, Hsinchu, Taiwan; ^4^Center for Intelligent Drug Systems and Smart Bio-devices (IDS2B), National Yang Ming Chiao Tung University, Hsinchu, Taiwan

**Keywords:** foodborne pathogens, biosensor, rapid detection, limit of detection, portable

## Abstract

Traditional foodborne pathogen detection methods are highly dependent on pre-treatment of samples and selective microbiological plating to reliably screen target microorganisms. Inherent limitations of conventional methods include longer turnaround time and high costs, use of bulky equipment, and the need for trained staff in centralized laboratory settings. Researchers have developed stable, reliable, sensitive, and selective, rapid foodborne pathogens detection assays to work around these limitations. Recent advances in rapid diagnostic technologies have shifted to on-site testing, which offers flexibility and ease-of-use, a significant improvement from traditional methods’ rigid and cumbersome steps. This comprehensive review aims to thoroughly discuss the recent advances, applications, and limitations of portable and rapid biosensors for routinely encountered foodborne pathogens. It discusses the major differences between biosensing systems based on the molecular interactions of target analytes and biorecognition agents. Though detection limits and costs still need further improvement, reviewed technologies have high potential to assist the food industry in the on-site detection of biological hazards such as foodborne pathogens and toxins to maintain safe and healthy foods. Finally, this review offers targeted recommendations for future development and commercialization of diagnostic technologies specifically for emerging and re-emerging foodborne pathogens.

## Introduction

Microbiological testing of foods is a fundamental part of food safety management. Farmers, food processors, and food safety regulatory agencies rely on microbial analysis for investigation, surveillance, and data analysis to accurately determine emerging risks (Jasson et al., 2010; Fleetwood et al., 2019). Due to the limitations of traditional microbiological methods, researchers have redirected their focus and resources to developing rapid, highly accurate, and reliable foodborne pathogen detection tools.

Conventional foodborne pathogen detection methods typically undergo both an enrichment process and selective microbiological plating before testing with immunological (i.e., antigen and antibody reactions), biochemical assays, and nucleic acid-based amplification such as conventional polymerase chain reactions (PCR) or quantitative PCR and loop-mediated isothermal amplification (LAMP). These conventional culture methods remain in use because they are harmonized and viewed as concrete, trusted systems in food diagnostics. However, while these culture methods may be performed with economical instruments and consumables, they are often tedious to perform, demand a substantial amount of resources such as liquid and solid media and reagents, and necessitate time-consuming processes and data collection methods. PCR methods are typically quick (3–6 h) and sensitive but demand intensive nucleic acid extraction techniques along with relatively costly equipment, while immunological assays maintain a significantly lower sensitivity (10^3^–10^5^ colony-forming unit or CFU/ml; Wang et al., 2017). Traditional methods are not suited for high-throughput screening of large food samples for the presence of one or multiple foodborne pathogens (Jasson et al., 2010; Hegde et al., 2012; Panwar et al., 2022). As a result, a robust, inexpensive, and rapid detection system, like on-site and portable biosensors, is needed to guarantee consumer food safety. This comprehensive review aims to thoroughly discuss the recent advances, applications, and limitations of portable and rapid detection technologies for routinely encountered foodborne pathogens and offer targeted recommendations for developing future diagnostic tools specifically for emerging and re-emerging foodborne pathogens.

## Rapid and portable screening and detection methods

Recently, a growing movement to push away from centralized laboratory sample processing and testing has led to the development of affordable and on-site detection systems (Mustafa et al., 2017; Silva et al., 2020). The World Health Organization (WHO) has established the necessary features of an acceptable rapid test in areas with limited resources with the acronym, ASSURED (Affordable, Sensitive, Specific, User-friendly, Rapid and Robust, Equipment-free, and Delivered to those who need it; Ben Aissa et al., 2017; Puiu et al., 2021). Previous research has separated rapid detection methods into three groups, namely (1) immunological-based, (2) nucleic acid-based methods, and (3) biosensors (Vanegas et al., 2017).

Immunoassays such as the commonly used enzyme-linked immunosorbent assay (ELISA) and agglutination kits for foodborne pathogen detection are relatively easy to perform but frequently generate false-positive results and are incapable of determining cell viability (Bhardwaj et al., 2017; Wang et al., 2017). Although PCR-based methods can increase the sensitivity of immunoassays approximately 100-fold, they require thermocycling equipment, trained personnel, and reliable infrastructures, which can be impossible to obtain in areas with few resources (Ben Aissa et al., 2017). Molecular or nucleic acid amplification techniques also require the need to break up cells, which becomes a limiting factor when working with rare cells that require more than a single test (Bole and Manesiotis, 2016). This is opposed to biosensor technology, which has been rigorously studied as a rapid, sensitive, and reliable tool, and can be utilized in real-time applications as it can integrate single or numerous laboratory functions allowing minimal sample preparation and superior detection on the same platform (Valadez et al., 2009; Kotsiri et al., 2022).

Biosensors are portable devices comprised of probes that integrate a biological element with an electronic component (transducer) to translate and generate a quantifiable signal (Naresh and Lee, 2021; Wang et al., 2022c). Biosensors are capable of detecting, recording, and transmitting information on the physiologic change and presence or absence of biological and chemical materials in specific environments. One of the main features of a biosensor is its ability to detect and measure even at low concentrations of molecules, specific pathogens, toxins, and other analytes in a relatively shorter turnaround time as compared to conventional methods. More importantly, biosensors require only small volumes of samples for analysis which makes it efficient and convenient, especially in areas where a large amount of samples needs to be tested immediately. A typical biosensor is comprised of bioreceptors, transducers, electronics, displays, and analyte.

As a result of the inherently long turnaround time of traditional pathogen detection methods, biosensors are designed, in an economical manner, to greatly reduce the required processing time between sample uptake and test results. As an analytical device, a biosensor has recognition elements that can be biological materials or their derivatives and/or other molecules that can mimic natural bioactive molecules for recognition (Lazcka et al., 2007). Recognition materials that are immobilized and anchored onto various platforms or transducers come in contact first with target analytes before the biosensing systems can generate signals. These molecules, such as bioligands and biocatalysts, contribute to the biosensors’ sensitivity and specificity, especially in diagnostic applications.

It is important to recognize, however, that the continuous advent of rapid pathogen detection technologies requires an in-depth understanding of the major differences in target analyte molecular interactions and biorecognition agents between devices (Vanegas et al., 2017).

## Portable biosensors classified based on bioreceptors or capture elements

Biosensors may use antibodies, aptamers, peptides, bacteriophages, and whole cells as capture elements or bioreceptors. Binding events occurring between the target of interest and receptors are converted into measurable signals facilitated *via* various transducers, such as impedance spectroscopy, cyclic voltammetry (CV), electronic field effects, potentiometry, amperometry as well as optical and thermal read-out principles (Eersels et al., 2016). Table 1 displays six types of recently developed biosensors for foodborne pathogens detection classified *via* capture elements (CE); (1) antibody, (2) aptamer, (3) amino acid, (4) antimicrobial peptides, (5) bacteriophage, (6) cells, and (7) biomimetic. The set of non-covalent interactions between recognition elements and target analytes determine the basis for a specific range of biosensing applications.

**Table 1:** Recently developed biosensors for foodborne pathogens detection.

Biosensors		**Target pathogens/molecules**	**Sample matrices**	**Time of analysis**	**Detection limit**	**Advantages**	**Disadvantages**	**References**
**Capture elements**	**Transducer/techniques**							
Antibody	Chemiluminescence	*Salmonell*a spp.	Milk, chicken multiple food	8 h	1 CFU/25 ml or g	Detection of viable/proliferative Salmonella spp. cells	Multisteps approach can be cumbersome; requires highly skilled staff	Li et al. (2019)
		*Salmonell*a spp.	Samples milk	6–8 h	3.63 × 103 CFU/ml	Use of phage mediation enhanced sensitivity	Non-portable; not for on-site use	Zhang et al. (2022)
		Quinolone			0.022–0.065 μg/L	Reduced cost and shorter enrichment period (1.25 h)	Non-portable; not for on-site use	Yu et al. (2022)
	Colorimetric—LFA	*E. coli O157:H17* *S. Paratyphi A* *S. Paratyphi B* *S. Paratyphi C* *S. Enteritidis* *S. Typhi* *S. Choleraesius* *V. cholera O1* *V. cholera O139* *V. parahae-molyticus*	279 food samples (dairy and marine products, beverages, snacks, and meats)	20 min	104 or 105 CFU/ml (no enrichment); 10 CFU/0.6 mg (with enrichment)	Short turnaround time (20 min) if no enrichment; Portable/On-site	High false positive rate and high detection limit	Zhao et al. (2016)
		E. coli O157:H17	Ground beef and spinach samples	—	104–105 CFU/ml	On-site results, low-cost analysis, and ease of use	High detection limit	Jung et al. (2020)
		*E. coli* O157:H17	Water, watermelon, milk, beef	3 h	103 CFU/ml (pure culture); 104–106 CFU/ml (complex matrices); 1 CFU/25 g (with enrichment)	Less expensive than traditional captureantibody dependent LFA	High detection limit; High rate of false negative (no Control Line)	Wu et al. (2022)
	Electrochemiluminescence (ECL)	*L. monocytogenes*	Milk, sausage, and ham samples	—	0.104 × 10^−1^ CFU/ml	Low detection limit	Cannot distinguish live from dead cells	Jampasa et al. (2021)
	Electrochemical—Differential pulse voltammetry	*S. aureus*	Pure culture, milk	1 min	28.55 CFU/ml (blank/pure culture); 1 × 10^4^–1 × 10^10^ CFU/ml (milk)	Short turn-around time (1 min)	High detection limit	Wu et al. (2021)
		*S. aureus*	Milk	—	2 CFU/ml	Low detection limit	Cannot distinguish live from dead cells	Wang et al. (2022b)
	Optical fiber	*S. aureus*		30–40 min	3.1 CFU/ml	Low detection limit (inactivated *S*. *aures*)	Short shelf life	Chen et al. (2020)
	Quartz crystal microbalance (QCM) immunosensor	*E. coli* O157:H7	PBS; milk	4 h	23 CFU/ml (PBS); 53 CFU/ml (milk)	Low detection limit	Relatively long turn-around time	Shen et al. (2011)
		*E. coli* O157:H7	Wild blueberries	18 h	0–99 CFU/ml	Enrichment and detection of viable cells in one system	Portability issue due to the enrichment system	Guo et al. (2012)
		*C. jejuni*	Pure culture	—	150 CFU/ml	Low detection limit	Cannot distinguish live from dead cells	Masdor et al. (2016)
		*S.* Typhimurium	Chicken meat	approx. 2 h with enrichment	10^0^ CFU/ml	Short turn-around time; Low detection limit	Portability issue due to the enrichment system	Fulgione et al. (2018)
	Surface acoustic wave (SAW)	*Salmonella* spp., *B. cereus*, *Listeria* spp., and *E. coli*	Milk	4.5 h (3 h enrichment)	1–5 cells/25 ml (*S*. Typhimurium)	Enrichment and detection of viable cells	Requires cumbersome pre-treatment steps; not validated for solid samples	Tsougeni et al. (2020)
		*E. coli* K12	Pure culture	---	10^5^ – 10^6^ CFU/ml	Expandable to other applications	High cost and high detection limit	Lamanna et al. (2020)
	Surface Plasmon Resonance (SPR)	*Salmonella* spp. and STEC strains	Chicken carcass rinse	Overnight enrichment	10^6^ CFU/ml (*Salmonella* spp.) and 1 CFU/ml (*Salmonella* spp. with enrichment)	Simultaneous detection of multiple target bacteria; enrichment ensures detection of viable cells	High cost and high detection limit if no enrichment step	Park et al. (2021)
		*S.* Typhimurium	Buffer and romaine lettuce	200 min	4.7 log CFU/mL (buffer) and 5.2 log CFU/g (romaine lettuce)	Stable, high surface-to-volume ratio due to MNPs	Multiple steps, high cost and high detection limit	Bhandari et al. (2022)
Aptamer	Quartz crystal microbalance (QCM)	*E. coli* O157:H7	Pure culture	50 min	1.46 × 10^3^ CFU/ml	Short turn-around time	Assay was only tested in pure culture, may need longer assay time with potential pre-treatment steps during in real applications.	Yu et al. (2018)
	Colorimetric – LFA	*E. coli* O157:H7	Pure culture; milk		7.6 × 10^1^ CFU/ml (pure culture) and 8.35 × 10^2^ CFU/ml (milk)	Portable, low detection limit	Cannot distinguish live from dead cells	Ren et al. (2021)
	Optical fiber	*E. coli* O157:H7 (heat-killed)	Buffer solution		10 CFU/ml	Real-time measurement; samples at picoliter levels	Prone to cross-reactivitity	Janik et al. (2021)
	Evanescent wave dual-color fluorescence	*E. coli* O157:H7 *S.* Typhimurium	Juice, tap water, and wastewater	35 min	340 CFU/ml (*E. coli* O157:H7) and 180 CFU/ml (*S.* Typhimurium)	Short turn-around time, simultaneous detection of multiple pathogens	Prone to cross-reactivitity, portability issues	Fang et al. (2021)
Amino acid	Gold sensor/Whole cell imprinting, SPR and QCM	*E. coli*	Water	7 min (QCM) and 20 min (SPR)	1.54 × 10^6^ CFU/ml (SPR); 3.72 × 10^5^ CFU/ml (QCM)	Short turn-around time	Prone to cross-reactivitity	Yilmaz et al. (2015)
	Gold electrode/ Microcontact imprinting, capacitive biosensing	*E. coli*	River water		70 CFU/ml	Real-time measurement	Prone to cross-reactivitity	Idil et al. (2017)
	Impedimetric	*S. aureus*	Milk	10 min	2 CFU/ml (pure culture) and 10^3^ (milk)	Short turn-around time	Pre-treatment of samples needed	Wang et al. (2021)
Anti-microbial peptides	Microfluidic chip	*E. coli*	Pure culture	< 30 min	10^3^ cells/ml	Short turn-around time	Prone to cross-reactivitity	Yoo et al. (2014)
Bacterio-phage	Potentiometric	*L. monocytogenes*	Coastal sea water	1 h	10 CFU/ml	Short turn-around time	Prone to cross-reactivitity	Lv et al. (2018)	Polyethylenimine (PEI)-carbon nanotube (CNT)/Impedimetric	*E. coli* B	Pure culture	---	1.5 × 10^3^ CFU/ml	Inexpensive alternative to antibodies	Prone to instability	Zhou et al. (2017)
		Luminescence	*E. coli*	Water	3 h with enrichment	< 10 CFU/ml	Short turn-around time, live cells are detected	Prone to cross-reactivitity	Hinkley et al. (2018)
	Amperometric	STEC O26, O157, and O179	Fresh ground beef and pasteurized apple juice	< 1 h	10–10^2^ CFU/g or ml	Short turn-around time, live cells are detected	Prone to instability	Quintela and Wu (2020)
	Electrochemical	*E. coli*	Spinach leaves	6 h with enrichment	1 CFU/ml	Inexpensive alternative to antibodies	Prone to instability	El-Moghazy et al. (2022)
	Biolayer interferometry (BLI)	*S. aureus*	Ice cube and light soy sauce	12 min (binding time)	13 CFU/ml	Live cells are detected	Portability issues	Liu et al. (2022)
	Polymerase Chain Reaction (PCR)	*S.* Typhimurium	Milk, lettuce	< 3 h	7 CFU/ml	Short turn-around time	Costly and cumbersome steps	Wang et al. (2022a)
Mamma-lian cells	Human ileocecal adenocarcinoma cell line (HCT-8) and immune-fluorescence	*S.* Enteriditis	Milk, ground chicken, cake mix, and eggs	10–12 h with enrichment	10^5^–10^8^ CFU/ml	Novel and can be adopted to other applications	Costly, cumbersome steps, high detection limit	Xu et al. (2020)
Biomimetic materials	Colorimetric—M13 bacteriophage	Volatile organic compounds and trinitrotoluene (TNT)	Gas phase	---	300 ppb	Highly portable	Prone to cross-reactivitity	Oh et al. (2014)
	Heat transfer method (HTM)—surface-imprinted polyurethane layers receptors	*E. coli*	Buffer, apple juice	---	100 CFU/ml	Portable, low detection limit	Prone to cross-reactivitity, cannot distinguish live from dead cells	Cornelis et al. (2019)
	Fluorescence—Teicoplanin	*S*. *aureus* and *L*. *monocytogenes*	PBS buffer solution, spinach, and ground beef	<2 h	10 CFU/ml (PBS and juice) and 10^2^ CFU/ml (spinach and ground beef)	No pre-treatment steps needed, short turn-around time	Cannot distinguish live from dead cells	Chen et al. (2022)
	Heat transfer method (HTM)/impedance—polydimethylsilo-xane (PDMS) films	*E. coli*	Strawberry-watermelon juice	—	80 ± 10 CFU/ml	Low cost	Cumbersome steps, cannot distinguish live from dead cells	Arreguin-Campos et al. (2022)

Groupings are based on the six types of capture elements (CE); (1) antibody, (2) aptamer, (3) amino acid, (4) antimicrobial peptides, (5) bacteriophage, (6) cells, and (7) biomimetic. The non-covalent interactions between the recognition elements and ligands dictate the basis for a specific range of biosensing applications.

## Antibodies-based biosensors

Because of their high affinity and specificity to targets, antibodies are commonly incorporated as CEs in biosensors (Ertürk and Lood, 2018). Additionally, antibody binding fragments are relatively easy to construct *via* protein engineering and are widely used in nanotechnology applications (Trilling et al., 2014). Various foodborne pathogens like Shiga toxin-producing *Escherichia coli* (STEC) O157:H7, *Salmonella* spp., *Campylobacter* spp., *Vibrio* spp., and viruses have been detected *via* antibody-based biosensors paired with a wide variety of transducers and techniques such as chemiluminescence (Li et al., 2019; Yu et al., 2022; Zhang et al., 2022), colorimetric (Zhao et al., 2016; Jung et al., 2020; Min et al., 2021; Wu et al., 2022), electrochemiluminescence (Jampasa et al., 2021), biomimetic (Wu et al., 2021), electrochemistry (Wang H. et al., 2022), optical fiber (Chen et al., 2020), quartz crystal microbalance (QCM) immunosensor (Shen et al., 2011; Guo et al., 2012; Masdor et al., 2016; Fulgione et al., 2018), surface acoustic wave (SAW; Lamanna et al., 2020; Tsougeni et al., 2020), and surface plasmon resonance (SPR; Park et al., 2021; Bhandari et al., 2022).

Chemiluminescence is generally generated by redox reactions wherein excited electrons release photons as it returns to a ground state (Yu and Zhao, 2021). A detection technology that employed a combination of immunomagnetic separation and chemiluminescence system [HRP-catalyzed luminol-H_2_O_2_ with 4-(1-imidazolyl)phenol] was able to detect proliferative cell(s) of *Salmonella* spp. strains in food samples (milk or chicken; Li et al., 2019). It achieved a 1 CFU/25 ml or g LOD but with a two-step enrichment approach (8 h). This multi-step and cumbersome approach necessitates a highly-trained staff to perform, which can be disadvantageous to laboratories with a high volume of samples and limited staff. However, with the inclusion of an efficient enrichment protocol, the approach was able to distinguish proliferative or viable cells apart from dead cells. Similarly, a double nanobody sandwich chemiluminescent enzyme immunoassay with bacteriophage mediation reported a LOD of 3.63 × 10^3^ CFU/ml (*S*. Typhimurium) in multiple food samples after 6–8 h incubation of fewer than 10 bacterial target cells (Zhang et al., 2022). The chemiluminescence method in the assay attempted to replace the conventional chromogenic reaction, and the bacteriophage mediation further enhanced the sensitivity. However, the need for a chemiluminescence intensity reader (i.e., microplate reader) poses the issue of portability for on-site detection, which limits the use of the technology. A fully automated chemiluminescence and optical-based immunosensor which also incorporated a pre-treatment step and immunomagnetic beads separation prior to chemiluminescent/optical fiber sensing, detected quinolone within the range of 0.022–0.065 μg/L in milk samples (Yu et al., 2022). The combination of labeled antibodies and chemiluminescent substrates efficiently yields a signal that is proportional to the amount of target analytes, but the required pre-treatment steps still pose a challenge in carrying out a fully automated and portable device for end-users.

In terms of portability, an immune-based colorimetric approach such as the lateral flow assay (LFA) that transduces Ab-antigen binding onto a color change in an on-site handheld platform, has become widely popular and available. Up-converting phosphor (UCP) particle (UPT) has been previously incorporated onto a LFA system (Zhao et al., 2016). The UPT-LFA method is formulated on anti-Stokes shift optical properties and on the highly stable fluorescence of UCP (Yan et al., 2006). Construction of individual UPT-LFA strips was based on Ab-sandwich binding affinity on the multi-channel UPT-LFA system. Ten foodborne pathogens, including STEC O157:H17, *S.* Paratyphi A, *S.* Paratyphi B, *S.* Paratyphi C, *S.* Enteritidis, *S.* Typhi, *S.* Choleraesius, *V. parahaemolyticus, V. cholera* O1, and *V. cholera* O139 were simultaneously detected from 279 samples. The sensitivity of the technology was in the range of 10^4^ or 10^5^ CFU/ml without enrichment, which improved to 10 CFU/0.6 mg post enrichment (14 h) and with a turnaround time of 20 min. However, there are recognized limitations, such as the inclusion of multiple lanes in a strip having a high chance of false binding as well as signal encoding, which would require several adjustments of its components, i.e., optical source, receiver, and filter, further adding complexity to the system. Additionally, the interpretation of color change can also cause uncertainty. To increase accuracy, commercially-available LFA systems for direct quantification of *E. coli* O157:H7 cells in ground beef and spinach samples (LOD, 10^4^–10^5^ CFU/ml) were attached to smartphone imaging systems *via* a high-resolution camera (Jung et al., 2020). These smartphone-based LFAs mainly rely on the computing power of smartphones without the need of enrichment. The use of smartphones and apps has allowed the detection of slight color changes in the test line, thus reducing ambiguity. Pre-treatment steps and signal amplifiers may still be supplemented to remove interferants and enhance signal transduction, respectively, hence improving sensitivity.

**Figure 1:**
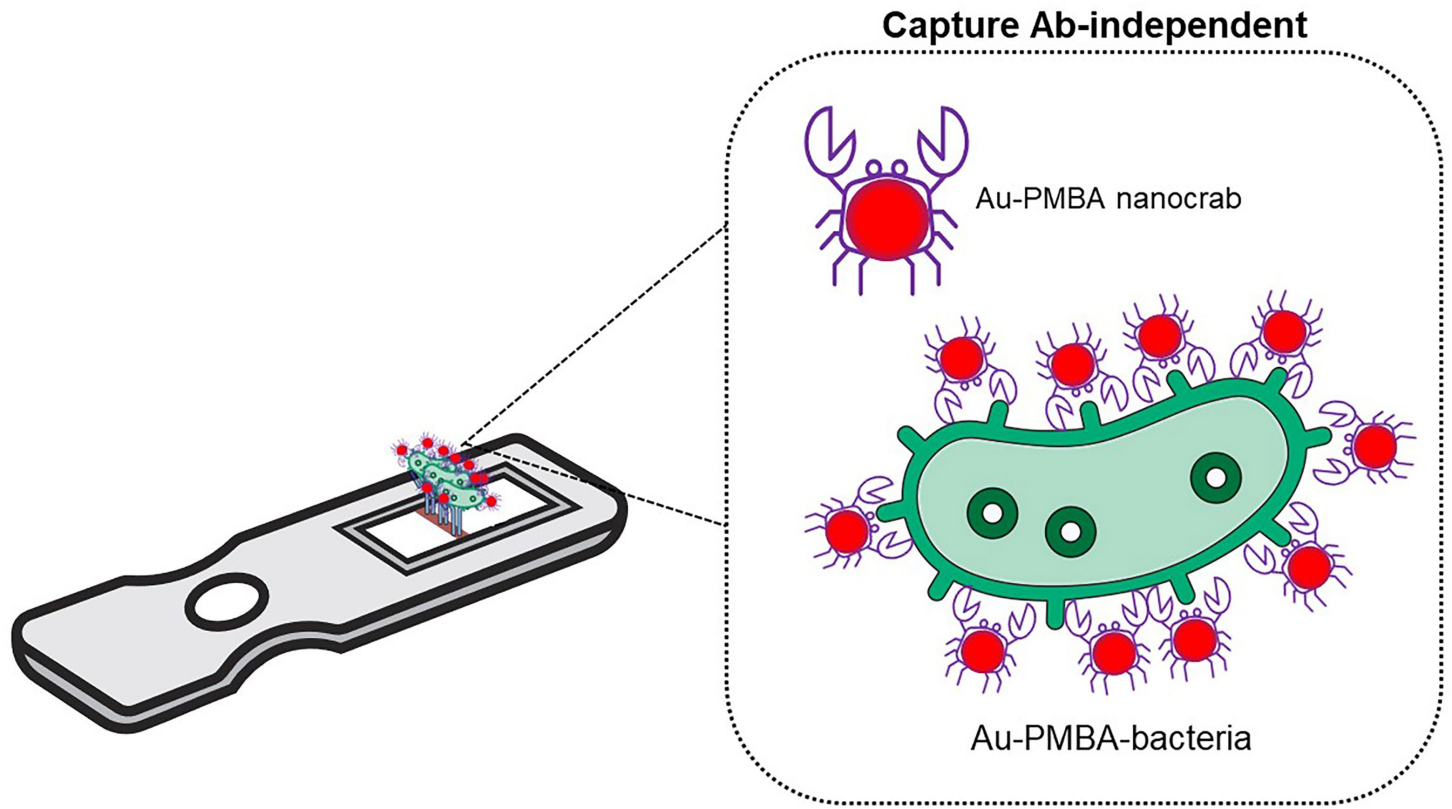
Lateral flow assay. A novel LFA with p-mercaptophenylboronic acid-modified AuNPs or “Au − PMBA nanocrabs” substituted the traditional LFA AuNP-labeled antibody (Wu et al., 2022).

Captured gold nanoparticles (AuNPs) on antibody-functionalized membranes dictate the sensitivity of LFA, and exploring new AuNP-conjugates for labeling target analytes is beneficial to enhance the platform’s sensitivity (Kim et al., 2016). An LFA integrated with p-mercaptophenylboronic acid-modified AuNPs or Au − PMBA nanocrabs, which substituted the traditional AuNP labeled antibody, has shown its ability to capture STEC O157:H7 with a LOD as low as 10^3^ CFU/ml in complex matrices (Figure 1; Wu et al., 2022). This LFA system which has a new strategy feature, allows universal bacterial binding for improved and rapid detection of such biological hazards. However, one of the major disadvantages of this approach is its inherently high false negative rates due to the absence of a control line in the LFA system.

Detection of a foodborne pathogen group can be more challenging relative to other outbreak-causing groups due to its rapid multiplication at ambient conditions such as *L. monocytogenes* (Shamloo et al., 2019; Jampasa et al., 2021). To respond to the safety needs of the food industry, an electrochemiluminescence (ECL) device integrated with nitrogen-decorated carbon dots, antibodies, and immunocomplexes on screen-printed carbon electrode for direct screening of *L. monocytogenes* was reported by Jampasa et al. (2021). At optimal conditions, the ECL device achieved a superior sensitivity of 0.104 CFU/ml in milk, sausage, and ham samples without enrichment, an improvement which can be attributed to the doped nitrogen and co-reactant used during ECL-resonance energy transfer (RET) process. This novel technology provides an environment-friendly approach due to the absence of heavy metal in the detection system as it utilized carbon dots instead of ruthenium or quantum dots. With its superior detection limit, the chance of detecting dead cells is relatively high due to the absence of short-enrichment steps.

A mussel inspired coating [ε-poly-L-lysine-3,4-dihydroxy benzaldehyde (EPD)]-based electrochemical immunosensor targeting *S. aureus* in drinks was recently reported (Wu et al., 2021).

Mussels are rich in catecholic amino acid, specifically 3,4-dihydroxyphenylalanine (DOPA), which has a strong wet adhesion property. Wu et al. (2021) used a biomimetic polymer EPD to fabricate a scaffold on a gold electrode surface, providing it with increased stability and antibody-binding ability due to its excellent wet adhesion features. More importantly, since the EPD exhibits pH-responsive properties, increased acidity allowed a cascading event *via* ε-poly-L-lysine (ε-PL) that ultimately killed *S. aureus*. Differential pulse voltammetry (DPV) suggested a LOD of 28.55 CFU/ml with a signal-to-noise ratio of 3 in pure culture and 1 × 10^4^–1 × 10^10^ CFU/ml range in milk samples. It had a short-turnaround time and achieved a significant bactericidal performance. Its high detection limit may pose a disadvantage to end-users, considering the complexity that comes with fabricating the system.

Immuno-based electrochemical biosensors are one of the most popular types of detection methods due to their well-characterized biological interactions (Setterington and Alocilja, 2012; Gao et al., 2017, 2018). Nanomaterials accelerate the transfer rate of electrons and increase the contact area of the electrodes on electrochemical biosensors allowing them to maintain efficient signal amplification (Pei et al., 2013; Hu et al., 2018; Feng et al., 2021). Dendritic mesoporous silica nanospheres (DMSNs) with Ab-silver sulfide quantum dots (Ag2S QDs) complexes were constructed and employed as signal amplification labels for electrochemical screening of *S. aureus* in milk samples (Wang H. et al., 2022). Due to the high loading capacity of Ag2S QDs, an individual bacterial cell was labeled, facilitating a superior immunosensor LOD of 2 CFU/ml in milk samples. This technology can be utilized as the initial screening method in the production lines due to its excellent detection limit but may suffer instability after several days (>15 days) due to the nature of Ab-conjugated Ag2S/DMSNs.

A label-free optical fiber biosensor based on a tapered single mode-no core single mode fiber coupler (SNSFC) structure functionalized with pig IgG for inactivated *S. aureus* detection has been reported by Chen et al. (2020). A wavelength shift of 2.04 nm was generated for a period of 30 min for the quantification of *S. aureus* (7 × 10^1^ CFU/ml) with a LOD of 3.1 CFU/ml. The study only tested *S*. *aureus* in its inactivated form, and the application of technology did not cover complex matrices. Further studies that would incorporate viable bacterial pathogens are needed to determine the accuracy and reliability of the proposed method. Moreover, the Ab-functionalized sensor had a relatively short shelf-life, 3 days at room temperature and 6 days in refrigerated temperatures (3–5°C), which would hinder its wider application and use.

Quartz crystal microbalance or QCM is a mass-based piezoelectric biosensor that recognizes and detects slight mass changes down to the nanogram level, resulting in a resonance frequency disruption that is directly proportional to the accumulated materials on the quartz surface (Deng et al., 2012; Guo et al., 2012; Yu et al., 2018). The quartz crystal resonator acts as the sensing component (Kimmel et al., 2011). These mass changes take place when the target analytes (e.g., whole cells of foodborne pathogens, toxins, and antigens) are captured and bound to the specific ligands immobilized on the surface (Deng et al., 2012). Shen et al. (2011) has previously reported an immuno-based QCM targeting STEC O157:H7, which utilized beacon immunomagnetic nanoparticles or BIMPs, streptavidin-Au, and an enrichment solution. The complex, STEC O157:H7-BIMPs, was loaded along with STEC O157:H7 polyclonal Ab (target antibody) and biotin-Ab (beacon antibody) to recognize, capture, and then separate whole STEC O157:H7 cells from the QCM setup. The LOD in phosphate buffer was 23 and 53 CFU/ml in milk samples. Guo et al. (2012) reported the first nanoparticle-functionalized piezoelectric biosensor-QCM immunosensor that offered simultaneous enrichment and detection of viable *E*. *coli* O157:H7 with a LOD of 0–99 CFU/ml within 18 h. This novel idea provides a semi-automated technique but may give rise to portabilility issues in terms of applying the system in multiple locations. In a separate study, a solution inoculated with *Campylobacter jejuni* was directly tested using a QCM platform with rabbit polyclonal antibody and achieved a LOD of 150 CFU/ml (pure culture; Masdor et al., 2016). Using QCM with 2 h of pre-enrichment steps, *S.* Typhimurium was detected in chicken meat achieving a LOD of 100 CFU/ml (Fulgione et al., 2018). QCM is highly versatile and allows the evaluation of various interactions between materials (Fee, 2013). In addition, QCM permits *in situ* monitoring of bacterial growth such STEC O157:H7 and *S. mutans* due to its relatively large surface area, which is significant in terms of short enrichment period and real-time detection (Schofield et al., 2007; Guo et al., 2012). The major challenges that are routinely experienced in using QCM biosensors include difficulty in regenerating the crystal surface, expensive packaging cost, and the complicated application of its fluidic system (Rocha-Gaso et al., 2009).

Surface acoustic wave or SAW biosensors can be used to screen proteins, sugars, nucleic acids, and viral structures. SAW biosensors yield and effectively detect acoustic waves through interdigital transducers (IDT), which can be found on the exterior surface of piezoelectric crystals. Under such conditions, the acoustic energy is limited around the exterior area of the device, thus falling within the scope of the acoustic wavelength. The resulting wave is highly sensitive to any variation on the surface due to changes in mass, conductivity, and viscosity (Länge et al., 2008). Four groups of bacterial pathogens (*Salmonella* spp., *B. cereus*, *Listeria* spp., and *E. coli*) were detected in a recently developed compact platform which was capable of immuno capture, lysis, DNA amplification, and integrated with an SAW sensor (Tsougeni et al., 2020). A pre-enrichment step (3 h) was needed prior to loading the samples onto the platform’s chip as well as a short centrifugation to concentrate the target analytes. The entire process (sample-to-answer) took 4.5 h, and based on the acoustic data, the LOD for *S.* Typhimurium was 1–5 cells/25 ml in milk samples. The highly integrated system was able to reduce the assay time by 80% as compared to similar technology. However, the cumbersome pre-treatment steps open up the possibility of contamination and require skilled staff to perform. Lamanna et al. (2020) fabricated an aluminum nitride (AIN)-based conformable SAW on recyclable polyethylene naphthalate (PEN) substrate to detect *E. coli* K12 *via* protein A/antibody, a novel approach for SAW which achieved a LOD of 10^5^–10^6^ CFU/ml. The merging of AIN and PEN into one system can potentially provide recyclable radio-frequency identification devices, which may include a water quality monitoring system and smart packaging, which require further optimization to improve its sensitivity.

The SPR phenomenon is an event in which incident light is absorbed by a surface at specific incident angle and wavelength (Wang et al., 2022b). Fluctuations in the molecular mass of the biomarker on the sensor surface cause a shift in the resonance wavelength or resonance angle, allowing quantitative real-time monitoring of the biomarker. A label-free immunoassay for screening *Salmonella* spp. and STEC strains on commercial chicken carcass rinse with SPR imaging (SPRi) was reported by Park et al. (2021). The SPRi biochip was functionalized with anti-*Salmonella*, anti-*E. coli* antibodies, and IgG controls. The LOD (*Salmonella* spp.) was 10^6^ CFU/ml for direct screening, which was then improved to 1 CFU/ml when coupled with an overnight enrichment. With pre-treatment steps, the sensitivity of SPR immuno-platform usually improves up to several magnitudes.

A magnetic nanoparticle-enhanced SPR biosensor was recently used for the detection of *S*. Typhimurium in romaine lettuce (Bhandari et al., 2022). This method included mAbs specific to the flagellin of *S*. Typhimurium bound to superparamagnetic nanoparticles (MNPs; 50 nm) and recovery of cells using vacuum filtration. With no enrichment step, the LOD was reported to be 4.7 log CFU/ml in buffer and 5.2 log CFU/g in romaine lettuce using the pre-incubation sandwich assay (one-step) approach. The combination of MNPs and flagellin-specific monoclonal antibodies effectively amplified the signal; however, the detection limit is still relatively high as compared to other detection methods.

Antibody-based biosensors commonly encounter cross-reactivity with non-target bacteria which can yield false positive results. Immunosensors without enrichment cannot differentiate viable, damaged, or non-viable bacterial cells because antibodies can still recognize and actively bind to the antigens that are present even in dead bacterial cells (Tlili et al., 2013). Antibodies often require intermediate protein and are dependent on non-covalent protein–protein interactions, which shortens the lifespan of the immunosensor. Moreover, storage temperatures need to be controlled to prevent denaturation, which can affect the reliability of immunosensors for routine on-site analysis.

Live animals are required in the production of monoclonal antibodies. Immune response is elicited to produce the desirable antibodies; thus, its production is highly reliant on the environmental and health conditions of the hosts. The maintenance of the animal facility directly contributes to the high production cost of antibodies; thus inexpensive non-immuno options for both recognition and binding element to capture target analytes should be explored.

## Aptamer-based biosensors

Aptamers are receptors that are evolved by systematic evolution of ligands by exponential enrichment or SELEX to bind to target proteins, cells, or other molecules of interest (Balamurugan et al., 2008; Wang et al., 2019). The most commonly used aptamers are DNA and RNA sequences which usually undergo certain modifications to enhance biocompatibility with the sensing platforms. In SELEX, target materials are incubated with a random single-stranded DNA (ssDNA) library pool. Those unbound sequences are removed by washing, while bound sequences are efficiently recovered. Positive controls are incubated with the recovered sequences for purification and elimination of non-specific sequences. The recovered sequences are further amplified and enriched by PCR for target binding. During PCR, labeled primers, i.e., fluorescein isothiocyanate (sense) and biotin (antisense) are used to amplify bound sequences. The antisense strands products are also eliminated to generate ssDNA in the succeeding rounds of selection.

Shiga toxin-producing *Escherichia coli* O157:H7 was previously detected by using an aptamer-based QCM biosensor (Yu et al., 2018). To generate highly specific aptamer sequences, the authors conducted 19 rounds of selection with STEC O157:H7. For counter selection, six rounds with non-target bacteria including *L. monocytogenes, S. aureus,* and *S.* Typhimurium were performed to ensure non-cross reactivity. The QCM aptasensor achieved a sensitivity of 1.46 × 10^3^ CFU/ml for STEC O157:H7 with a 50 min assay time. It is important to conduct a robust SELEX procedure that would allow aptamer products with high affinity toward their target molecules. The technology was only tested in pure culture setups, and it may need longer assay time with potential pre-treatment steps during real applications. In milk samples, STEC O157:H7 was detected using an aptamer-exonuclease III (Exo III)–assisted amplification-based lateral flow assay (Ren et al., 2021). Its hairpin sequence post enzymolysis was identical to the target ssDNA sequence, thus allowing signal amplification and achieving a LOD of 7.6 × 10^1^ CFU/ml in pure culture and 8.35 × 10^2^ CFU/ml in milk samples. The combination of LFA, Exo III, and a hairpin probe has significantly enhanced the signal and simplified the amplification process of target nucleic acids. However, depending on the target pathogens, future applications may need to add a short pre-treatment step to ensure that viable bacterial cells are detected and not dead cells and/or nucleic acids from non-proliferative cells.

Janik et al. (2021) took advantage of the microcavity in-line Mach-Zehnder interferometer (μIMZI) induced in an optical fiber wherein two highly specific peptide aptamers acted as the bioreceptors to detect *E. coli* O157:H7. One of the main advantages of this technology is that the functionalized biosensor surface allows the detection of changes in the layer down to the sub-nanometer level. The LOD demonstrated real-time measurements reached 10 CFU/ml of heat-killed *E. coli* O157:H7 in buffer solution. This application can be extended not only to other bacterial pathogens but also to emerging foodborne viruses.

Cy3-apt-E and Cy5.5-apt-S were used as fluorescence-labeled aptasensors mainly as biorecognition elements and reporters for *E. coli* O157:H7 and *S*. Typhimurium, respectively (Fang et al., 2021). The fiber nanoprobe etched nanopores were able to distinguish free aptasensors and aptasensors bound to pathogenic bacteria due to the limited penetrated depth of the evanescent wave and size difference between bacteria and nanopore. The LOD of *E. coli* O157:H7 and *S.* Typhimurium were 340 and 180 CFU/ml, respectively, in complex matrices (e.g., juice, tap, and wastewater). Due to the ease of use of the technology, advantages of aptamers, and real time detection capability, this new approach can be expanded to other complex matrices for initial screening of the most routinely encountered foodborne pathogens as well as environmental monitoring.

## Amino acid-based biosensors

**Figure 2:**
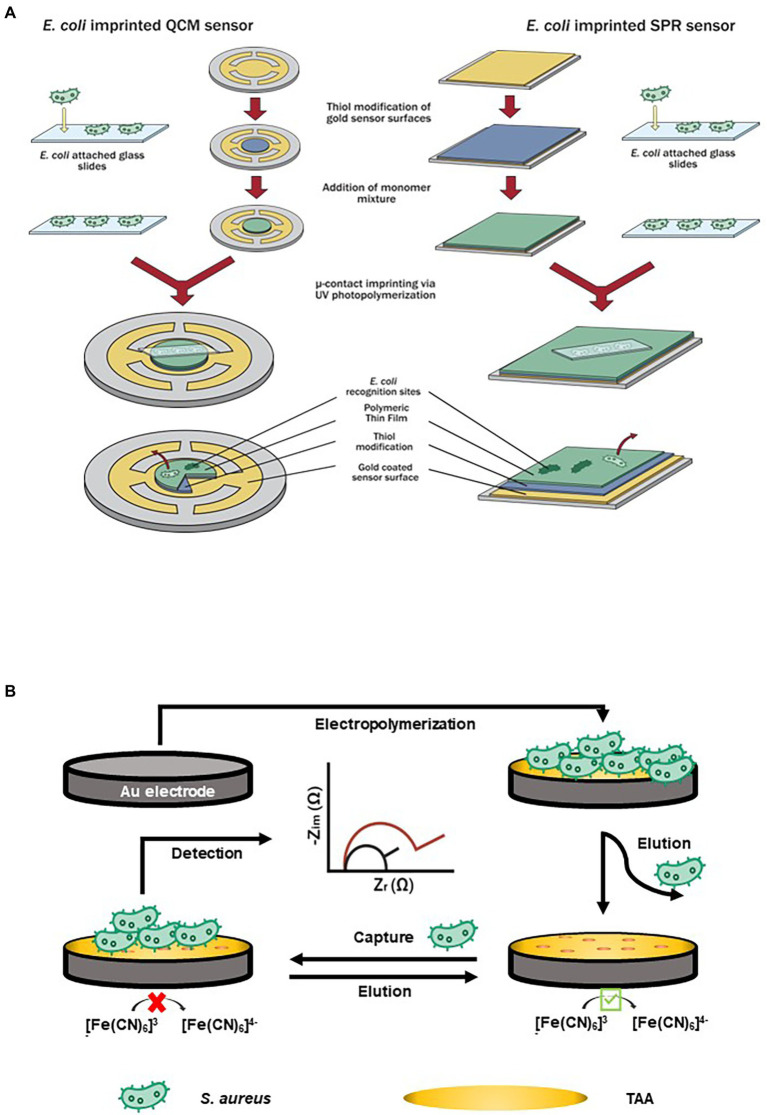
Examples of amino acid-based biosensors. **(A)** Schematic representation of microcontact imprinting. Microcontact imprinting of *E. coli* on QCM and SPR sensor surfaces. Image adapted from Yilmaz et al. (2015), and **(B)** Impedimetric sensor based on bacteria-imprinted conductive poly(3-thiopheneacetic acid; BICP) film for the rapid detection of *S. aureus* (Wang et al., 2021).

Synthetic forms of amino acids have been used and developed as recognition elements for micro imprinted biosensors. Yilmaz et al. (2015) reported a polymerizable form of histidine, N-methacryloyl l-histidine methylester or MAH, which can act similarly to antibodies in terms of recognizing and binding with whole bacterial cells (*E. coli*). Figure 2A shows a schematic representation of microcontact imprinting of whole bacterial cells (*E. coli*) with sensitive mass and optical-based devices. On the surfaces of SPR (optical) and QCM (mass) biosensors, the authors overlayed *E. coli* imprinted polymeric films and further tested its cross-reactivity toward non-target bacteria such as *Bacillus* spp. and *Staphylococcus* spp. A monomer solution was loaded onto the QCM and SPR surfaces prior to polymerizing it with *E. coli* layers. Polymerization was achieved by using UV light under a controlled nitrogen atmosphere. Due to the formation and availability of micro imprinting technology complementary cavities (bacterial stamps), chemical recognition of target *E. coli* on the surface sensor occurred upon its functionalization with MAH. Bacterial stamps were formed on the electrodes of the sensors allowing the recognition, binding, and capture of target *E. coli* in real-time application. This novel approach achieved a LOD of 3.72 × 10^5^ CFU/ml for QCM and 1.54 × 10^6^ CFU/ml for SPR technique.

The surface imprinting approach or SIP is a standard molecular imprinting technique that allows the assembly of its template solution on top of the substrate. As the template is extracted, high-affinity binding cavities are created where specific and only perfectly fitting targets can rebind (Eersels et al., 2016). Molecular imprinting technology or MIT creates artificial recognition and binding sites which match the shape, dimensions, and spatial orientation of its template (i.e.bacterial cells) and are then integrated into different transducers and platforms. Idil et al. (2017) developed an amino acid-based biosensor using both MAH and 2-hydroxyethyl methacrylate (HEMA) as monomers, and ethyleneglycol dimethacrylate (EGDMA) acting as its crosslinker *via* UV polymerization. The authors applied capacitive sensors for real-time monitoring and screening of target *E. coli* cells instead of SPR and QCM techniques, as previously mentioned. The LOD of the technology was 70 CFU/ml, and it could differentiate *E. coli* cells from non-target bacterial strains with similar morphological features.

Molecular imprinted polymers (MIPs) and MAH are both commonly incorporated onto amino acid-based biosensors. Artificial recognition pockets-like areas in polymeric media are formed by imprinting, which highly complement the morphologies (i.e., shapes, sizes, and spatial orientation) of target analytes and/or its functional groups (Ertürk and Mattiasson, 2017). Different biosensing platforms that are integrated with MIPs are an excellent option for detecting bacterial pathogens. This “molecular key and lock” approach, however, still has some areas that need improvement. Its detection limit is greatly affected by low binding capacity and the long turnaround time is a result of equilibration period between target analytes and binding materials. Moreover, the intricate cross-linking network slows down the diffusion of target molecules for specific binding and recognition. Follow-up studies and thorough investigation need to be conducted to identify the applicability, specificity, and sensitivity of MAH with other biological hazards, including outbreak-causing foodborne pathogens.

A bacteria-templated MIP named bacteria-imprinted conductive poly(3-thiopheneacetic acid) or BICP film on a gold electrode was used by Wang et al. (2021) to develop an impedimetric sensor for detecting *S. aureus* (Figure 2B). The BICP film had an original structure without cocci-shaped cavities on the polymer matrices, denoting that the imprinted sites were found at the polymer surface, hence allowing increased accessibility*. S. aureus* was detected within 10 min with low LOD of 2 and 10^3^ CFU/ml in milk samples.

## Antimicrobial peptides-based biosensors

**Figure 3:**
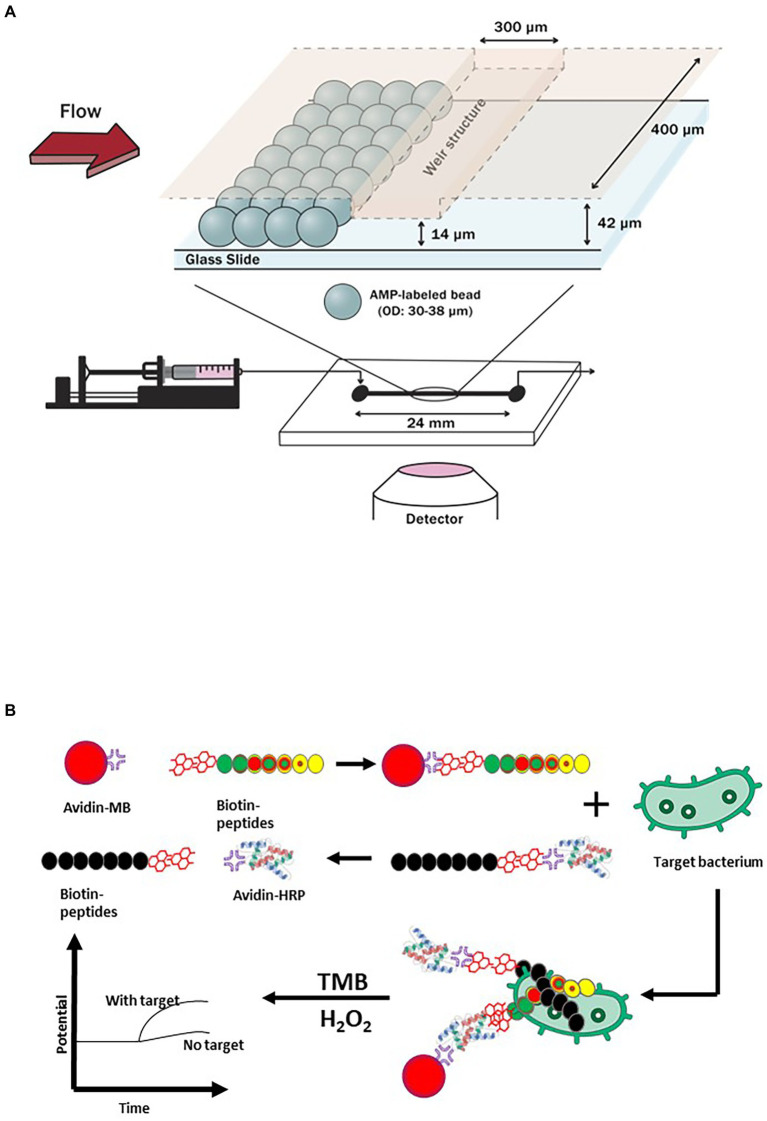
Examples of antimicrobial peptides (AMPs)-based biosensors. **(A)** A biosensing technique that targeted *E. coli* by utilizing a microfluidic chip with AMP (Magainin I)-labeled microbeads embedded on its channels (Yoo et al., 2014), and **(B)** A short antimicrobial peptide pair-based sandwich assay for potentiometric detection of *L. monocytogenes* (Lv et al., 2018).

Antimicrobial peptides or AMPs are excellent alternatives to antibodies for viral and bacterial pathogens detection. Yoo et al. (2014) developed a microfluidic chip coupled with magainin I-labeled microbeads specifically for detecting *E. coli* as shown in Figure 3A. Prior to embedding functionalized microbeads on the sensing channels, NH_2_ group and N-[γ-maleimidobutyryloxy] succinimide ester were used to modify its surface for AMP coupling and attachment. The recognition and capturing events of propidium iodide (PI)-stained *E. coli* took place due to the binding affinity of magainin I, an antibiotic and amphipathic peptide, with teichoic acid (TA) and lipopolysaccharide (LPS) found in the cellular membrane of *E. coli.* The technology was designed to allow propidium iodide-stained *E. coli* to flow into a microfluidic system that was connected to a high resolution fluorescence microscope. The increased flow rate of *E. coli* suspension resulted in a shorter turnaround time of 30 min to achieve a saturation level for the direct detection of *E. coli* cells. Cumulative changes in fluorescence intensity suggested that the LOD was 10^3^ CFU/ml.

Magainin I and other similar AMPs are excellent recognition elements for Gram-negative bacteria but can be challenging for Gram-positive bacteria due to the absence of LPS. When used with heterogenous samples, such as mixture of both Gram-negative and other enterobacteria, additional pre-treatment steps may be needed prior to conducting the assay to prevent cross-reactivity and non-specific binding. Moreover, conducting safety evaluation in terms of the potential health risks of routinely used AMPs toward humans, animals, and the environment is needed to prevent possible development of antibiotic resistance.

A short antimicrobial peptide pair-based sandwich assay was used for the potentiometric detection of *L. monocytogenes* (Lv et al., 2018). The novel AMP with a well-defined structure for *L. monocytogenes* was split into two fragments which acted as the peptide pairs for the sandwich assay (Figure 3B). With horseradish peroxidase used as a label, oxidation with hydrogen peroxide-induced a potential change on the electrode. The LOD of the assay was 10 CFU/ml. This approach can be adopted to detect other target pathogens or molecules; however, due to multiple binding sites, the rate of cross-reactivity can also increase.

## Bacteriophage-based biosensors

Significant advances in the development and use of bacteriophage or bacteriophage-derived molecules-based biosensors have occurred in recent years. Bacteriophages are natural and abundant biological entities that feature superior host selectivity, which is highly beneficial when used as recognition probes for bacterial pathogen detection (Singh et al., 2013). Bacteriophages can recognize and differentiate live or viable target bacterial cells and have a shorter turnaround time as compared to traditional cultivation techniques and the propagation of bacteriophages is relatively simple and inexpensive due to their widespread in nature (Hagens and Loessner, 2007).

**Figure 4:**
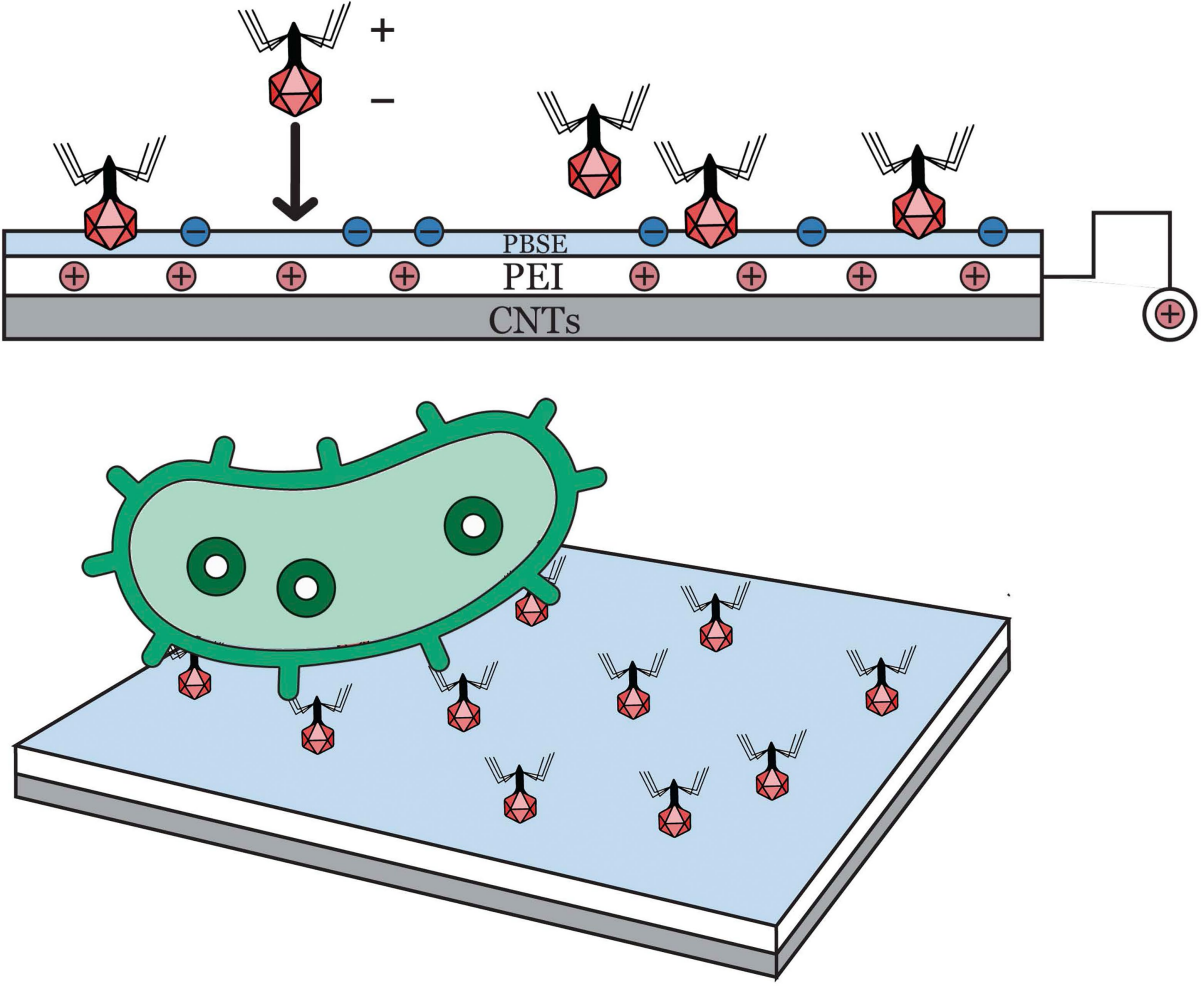
A new impedimetric biosensing based on carbon nanotube (CNT) with T2 bacteriophages for detection of *E. coli* B (Zhou et al., 2017).

Zhou et al. (2017) developed an impedimetric biosensor based on bacteriophage T2 and carbon nanotube (CNT) for screening *E. coli* B cells (Figure 4). Immobilized bacteriophage T2 on the surface of polyethylenimine (PEI)-modified CNT transducer and glassy carbon electrode served as the biorecognition element of the biosensing system. Initially, the platform’s surface was charge-enhanced rendering it appropriate for oriented bacteriophage immobilization by covalently linking its capsid on the surface. The charge-directed immobilization process was successfully conducted by applying a potential (+0.5 V vs. Ag/AgCl) to the working electrode for an hour (1 h). Bacteriophages, specifically tailed bacteriophages, have a net negative charge which allows immobilization *via* electrophoretic deposition as well as electrostatic interaction. Bacteriophage’s nucleic acids are exclusively localized in its head, maintaining a more negatively charge in that region and slightly positive charge along its tail fibers. Electrochemical impedance spectroscopy or EIS was used to monitor the capture of *E. coli* B cells, wherein the binding interactions between bacteriophage T2 and *E. coli* B cells resulted in interfacial impedance change. The LOD in pure culture setup was 3 log CFU/ml, which was relatively higher as compared to other electrochemical-based techniques. The results suggested that bacteriophages (T2) are highly effective recognition elements for detecting significant foodborne pathogens. An important note to consider is that the complex multilayer detection techniques may not be suitable for routine on-site or in-field testing.

A bacteriophage has been used as a reporter for the presence of *E. coli* in water (Hinkley et al., 2018). In this study, a genetically modified coliphage (T7) was used to express a luciferase (Nanoluc), which then acted as a bacterial contamination indicator. The Nanoluc reporter was fused with a crystalline-specific carbohydrate binding module. This novel work was able to concentrate the fusion reporter and achieve a LOD of <10 CFU/ml in 3 h. Quintela and Wu (2020) employed bacteriophages in a sandwich-type amperometric biosensor for the detection of STEC O26, O157, and O179 strains in complex matrices. Environmentally isolated bacteriophages were modified and directly immobilized onto a streptavidin-coated SPCE. Without the need for enrichment, the LOD was 10–10^2^ CFU/g or ml when applied to fresh ground beef and pasteurized apple juice. Similarly, a genetically engineered T7 bacteriophage encoding *pho* acted as a biorecognition element that activated overexpression of alkaline phosphatase during infection (El-Moghazy et al., 2022). The available alkaline phosphatase catalyzed electrochemical reactions that provided quantification of pathogenic *E. coli* on spinach leaves, achieving a LOD of 1 CFU/ml post enrichment.

Researchers have also explored utilizing bacteriophage-derived affinity molecules as recognition elements rather than the entire bacteriophage structures. Bacteriophage lysin (LysGH15) and long tail fibers have been recently incorporated as recognition elements to detect *S. aureus* and *Salmonella* spp., respectively (Liu et al., 2022; Wang et al., 2022a). LysGH15 lost its lytic activity through C54A mutation but retained its recognizing and binding ability to *S. aureus*. Liu et al. (2022) combined biolayer interferometry (BLI) with LysGH15, acting as its bioreceptor. The BLI-based method was able to detect *S. aureus* whole cells directly in food samples (ice cube and light soy sauce) with a LOD of 13 CFU/ml with a binding time of 12 min. Bacteriophage long-tail-fiber proteins (LTF4-a) were immobilized onto magnetic nanoparticles (MNPs) for specific separation and concentration of *S.* Typhimurium (Wang et al., 2022a). LTF4-a-MNP complex allowed the isolation and enrichment of *Salmonella* cells from artificially spiked food samples (milk, lettuce, and egg) prior to direct qPCR. The method achieved a LOD of ∼7 CFU/ml within 3 h.

Similar to other biosensors, the highly stable and effective immobilization of bacteriophages on biosensing platforms, either by passive physical absorption, chemical functionalization, genetic manipulation, and covalent binding to ensure properly oriented attachment plays an important role in the reliability, performance, sensitivity, and robustness of the biosensing systems. The successful immobilization of bacteriophages, which exposes its receptor binding domains for target recognition, and capture through strong inherent affinity to various receptors, should be complemented with the most appropriate biosensing techniques to generate the strongest signals with the least background noise.

The food and agricultural industries have widely accepted the use of rapid detection and screening techniques for spoilage organisms and outbreak-causing foodborne pathogens. However, improved and reliable testing technologies that allow reproducible same-day screening of initial low-level contaminants are required to respond to the industries’ needs (Leach et al., 2010; Wiedmann et al., 2014). With this, recognition elements such as bacteriophages have proven to possess enhanced biocompatibility, short reaction time, and superior features as an alternative to commonly utilized bioreceptors. Its abundance in nature, exclusivity with bacterial hosts, ease of propagation and modification, stability, especially in harsh conditions, are some of its advantages over other available recognition elements. However, most of the currently reported biosensors, either whole bacteriophage or bacteriophage-derived affinity molecules-based biosensors, have not achieved superior LOD in natural samples and complex matrices. This drawback can be attributed to the inefficient design of the biosensing system and the chosen platforms with inherent noise sources. The majority of these detection systems utilized a single-binding event between the capture element of the biosensors and the analyte(s) of interest. Bacteriophages have shown excellent specificity toward its bacterial host/analyte but its incorporation onto the detection technology and acting as biorecognition elements may need additional binding events to boost its sensitivity, reliability and specificity. A secondary binding event is commonly designed and utilized in a dual-site binding approach or sandwich assay. The initial element binds and captures the target molecule, while the secondary element serves as the reporter probe. The capture elements are usually immobilized onto the surface of solid substrates. Many bacteriophage-based biosensors are built and designed with screen-printed electrodes (SPEs). SPEs are highly flexible in terms of chemical modification, customization based on the required assembly, and great compatibility with other common platforms. SPE has a rapid test-to-results capability which provides an array of biosensing applications not only for foodborne pathogens but also other biological hazards such as toxins. The reporter probes are usually coupled with signaling molecules and moieties, which emit signals that are proportional to the amount of target analytes present in the reaction volumes. Bacteriophages have excellent biocompatibility with nanomaterials, fluorophores, enzymes, and other signaling moieties. By incorporating multi-layer recognition events, bacteriophage-based biosensors can achieve superior sensitivity specifically in detecting whole and viable bacterial cells, even when applied to complicated and highly complex samples and materials.

## Cell-based biosensors

Cell-based biosensors, or CBBs, primarily utilize live cells and appropriate transducers for detecting cellular physiological parameters, toxicity tests, rapid screening of microbial contaminants, as well as pharmaceutical effects (Banerjee et al., 2007; Liu et al., 2014). CBBs are by-products of tremendous research and advances in molecular biology, silicon microfabrication, and cell culture (Wang et al., 2005). CBBs are divided into two major parts: (1) the living cells which serve as the sensing element that receive and emit signals, and (2) a transducer that converts cellular and physiological output into quantifiable and verifiable signals. Similar to other biosensors, living cells as CBB’s receptors are isolated and immobilized on the surface of its accompanying transducer (Liu and Wang, 2009).

Xu et al. (2020) utilized a formalin (4% formaldehyde)-fixed human ileocecal adenocarcinoma cell line (HCT-8) to capture viable pathogens in combination with antibodies. *S*. Enteriditis was used to inoculate various food samples artificially. The LOD of the mammalian cell-based immunoassay (MaCIA) varied and influenced by the food matrix; 10^5^ CFU/ml in milk, 10^6^ CFU/ml in ground chicken, 10^7^ CFU/ml in cake mix, and 10^8^ CFU/ml in eggs. The food commodities used were representatives of products implicated in *Salmonella* outbreaks. The high protein, fat, or carbohydrate contents of the matrices greatly interfered with the assay resulting in poor signals and inferior sensitivity.

## Biomimetic-based biosensors

Surface chemistry and material science are increasingly shifting its attention to creating artificial matrices *via* biomimetic approach. Synthetic receptor strategies for both chemical detection and biosensing of specific analytes including proteins, viruses, and bacteria are rapidly transforming toward a robust and sustainable discipline (Hussain et al., 2013).

**Figure 5:**
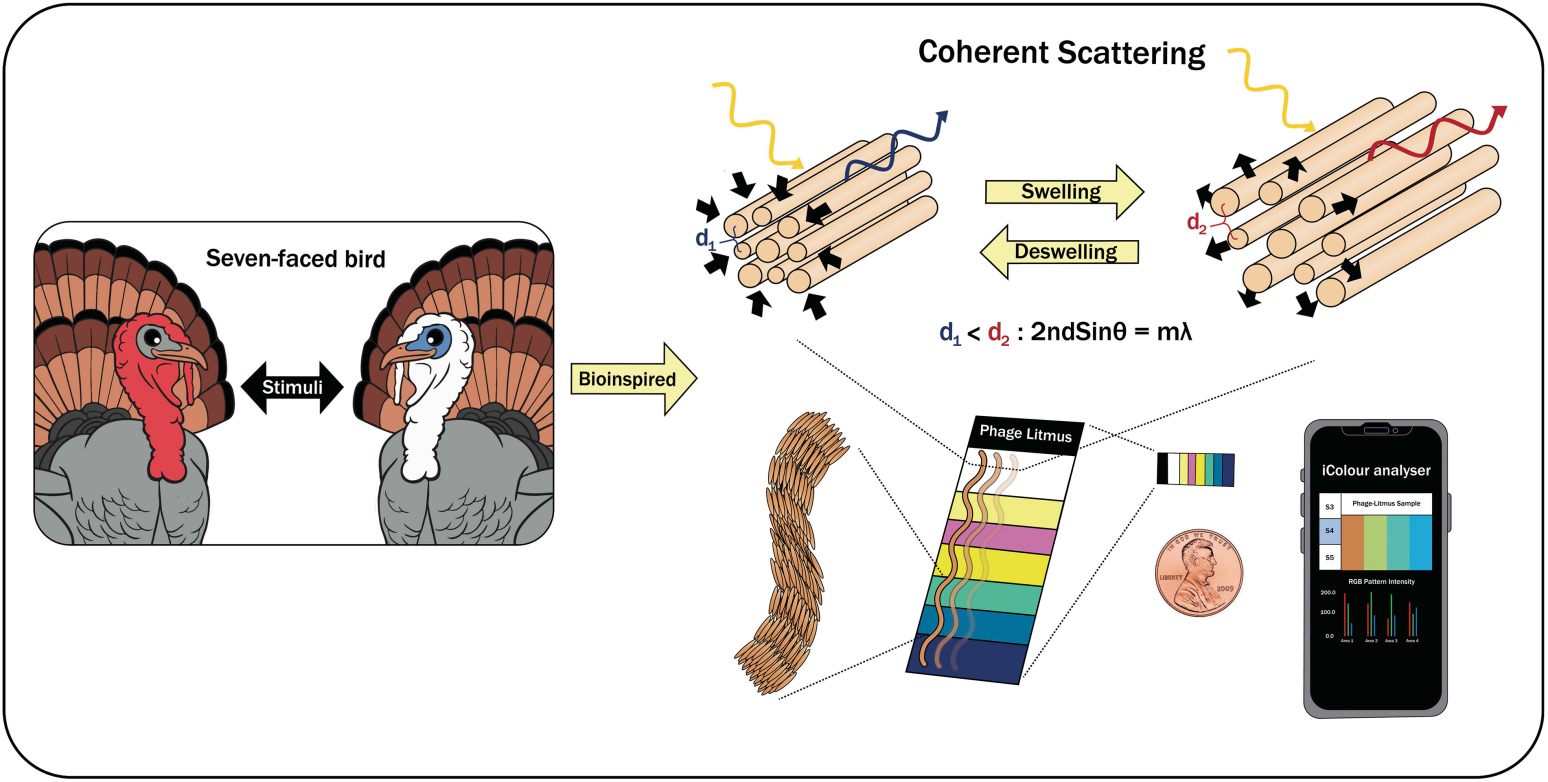
A biomimetic-based biosensor with Phage Litmus similar to collagen structures in turkey skin (Oh et al., 2014).

Oh et al. (2014) reported a biomimetic-based biosensor that was specific for volatile organic compounds (VOCs) and other important chemicals (e.g., trinitrotoluene—TNT; Figure 5). This colorimetric based-biosensor that they developed, called Phage Litmus, had phage-bundle nanostructures and viewing-angle independent color that mimic the collagen structures found in turkey skin. Upon exposure to different VOCs, Phage Litmus responds to fluctuations in humidity by rapid swelling and distinctive color changes. TNT (>300 ppb) was selectively distinguished over other chemicals by the efficient phage displaying binding motifs. The data that were collected was then analyzed by iColour Analyzer software that was installed in the smart phone allowing remote and on-site testing. The technology is a promising approach, and its application to non-gaseous materials needs to be explored and expanded.

A biomimetic sensor with surface-imprinted polyurethane layer receptors on stainless steel chips has been used to detect *E. coli* in apple juice samples (Cornelis et al., 2019). This technology measures the changes in thermal resistance between the chips and matrices during the target and capturing event. A meander structure acted as both a temperature sensor and a heater. The sensor achieved a 100 CFU/ml LOD for both phosphate buffered saline (PBS) solution and apple juice samples. Similarly, biomimetic dandelion-like magnetic nanoparticles have been recently used to capture and detect *S*. *aureus* and *L*. *monocytogenes* (Chen et al., 2022). Teicoplanin (Teic), a molecule that recognizes both Gram-positive and negative bacteria, was wired onto magnetic beads (MBs) *via* serum albumin (BSA) and PEG_2k_ forming biomimetic dandelion-like magnetic nanoparticles (MBs-PEG-BSA-Teic). The superior capture ability of MBs-PEG-BSA-Teic toward *S. aureus* (>93%) and *L. monocytogenes* (>86%) followed by separation and addition of fluorescence probes allowed the technology to achieve a LOD of 10 CFU/ml in PBS buffer solution and 10^2^ CFU/g in spinach and ground beef samples. This dual-mediated coupling approach significantly improved and enhanced the ability to capture bacterial pathogens in complex matrices without pre-treatment steps. Finally, an improved biomimetic thermal sensing of *E. coli* was recently reported by Arreguin-Campos et al. (2022), in which polydimethylsiloxane (PDMS) films were utilized as receptor layers and functionalized with graphene oxide (GO). The recognition and binding of the target onto the polymer resulted in a measurable change in temperature. This biomimetic sensor was applied to juice samples and achieved a LOD of 80 ± 10 CFU/ml which was superior to other thermal devices.

## Emerging biosensing technologies and approaches

Lab-on-chip (LOC) is an emerging and innovative solution that promotes moving of traditional detection assays into the point-of-care (POC) or in-field screening application. LOC efficiently integrates layers of laboratory capabilities and functions onto a handheld and portable platform, approximately 1 mm, with a highly intricate microfluidics system to fabricate miniaturized laboratories (Yoon and Kim, 2012).

Lab-on-chip as a CN-based immunoassay for screening of toxins such as *Staphylococcal* Enterotoxin B or SEB is a flexible approach designed to assist end-users in conducting testing in the field (Yang et al., 2013). Similarly, STEC O157 and *L. monocytogenes* were simultaneously screened and identified by a microfluidic duplex droplet digital PCR or ddPCR platform that employed a chip comprised of mineral oil-saturated polydimethylsiloxane (Bian et al., 2015). The authors used TaqMan-MGB fluorescent probes with the platform allowing it to detect at a single-molecule resolution level (10 CFU/ml) in artificially spiked drinking water (2 h). A lab-on-a-disk coupled with LAMP for the rapid detection of *Salmonella* spp. was investigated by Sayad et al. (2016).

The miniaturized system has allowed an all-in-one pathogen detection technology within 70 min in a microfluidic compact disk that included reagent preparation and target amplification *via* LAMP. The LOC achieved a 0.005 ng/μl DNA detection limit on tomatoes that were inoculated with *Salmonella* spp. These technologies offer flexibility in terms of testing samples outside the premise of laboratory with shorter turnaround time. It is important to consider that the amplification steps used in many LOCs cannot differentiate viable from non-viable or even injured cells, which is a key aspect in the field of food safety.

On-chip screening of *Pseudomonas aeruginosa* using IMS for isolation, light scattering technique, and machine learning strategy was also reported by Hussain et al. (2022). Its microfluidic system and photodetector collected the scattered light patterns and converted them into electrical signals. Four machine learning qualifiers trained and tested time-domain statistical features, which were then measured by its optical waveguides. *P. aeruginosa* was detected by the system with a LOD of 10^2^ CFU/ml within 10 min using the prepared samples.

Optical biosensors are detection technologies that can be easily used and judged simply by the naked eye. Nanomaterials such as gold and silver nanoparticles have SPR properties that efficiently and effectively play important roles in the detection process. Quintela et al. (2019) utilized oligonucleotide-AuNPs for the simultaneous detection of *Salmonella* spp. in food and environmental samples. To ensure detection of live bacterial cells, a short-enrichment step was incorporated prior to detecting target pathogens. With oligonucleotide-functional AuNPs, visible signals (LOD < 10 CFU/ml or g) were analyzed by using the naked eye, and no additional instruments were needed. Similarly, Marin et al. (2022) utilized aptamer-AuNP to detect *S. aureus* in powdered milk and infant formula. The analysis only took 30 min to perform and can be used on-site for rapid detection. Recently, nanozymes or artificial enzymes, which possess unique features for transducing signals, have been used for optical biosensor development. Recently, histidine-modified magnetic hybrid nanozymes have been developed by Wang et al. (2022d) as capture probes as well as signal amplifiers for the sensitive colorimetric detection of *S.* Typhimurium in food. This novel aptasensor with His-Fe3O4@Cu magnetic nanozymes achieved a LOD of 8 CFU/ml that is very applicable for on-site detection. Naked-eye detection simplifies the overall testing and allows untrained staff to conduct analysis remotely without the need of expensive equipment.

Smartphones are essentially used everywhere primarily to connect and communicate with other users. Due to the availability of its useful components (e.g., camera, software, battery, camera, display, and intuitive user interface) and wireless connectivity (e.g., Bluetooth and WiFi), smartphones are now converted into useful and reliable diagnostic tools (Rateni et al., 2017; Kanchi et al., 2018). These portable devices have been further developed, improved, and utilized in the healthcare sector, agricultural system and research, environmental monitoring, and military which provide opportunities for rapid and on-site detection of specific analytes (Rateni et al., 2017).

Shrivastava et al. (2018) developed a smartphone-based biosensor in combination with functionalized fluorescent magnetic nanoparticles for screening *S. aureus* in processed liquid matrices and samples. Fluorescent magnetic nanoparticles or FMNPs and smartphone imaging allowed for capturing and detection of *S*. *aureus*. A cassette was fabricated to accommodate various samples and allow its mixing with aptamer-conjugated FMNPs. The imaging of FMNP-tagged *S. aureus* was performed using a white light-emitting diode (LED) integrated in a smartphone camera. The LOD of the detection device was reported to be 10 CFU/ml as it counted individual *S. aureus* cells from food items. Since the peanut milk sample was the main food model used during the development, other food samples such as those with high protein/enzyme contents may reduce the sensitivity of the device as it can affect the recognition and binding of aptamers to its target sites.

Zeinhom et al. (2018) developed a smartphone-based immunosensor that was specific for the detection of STEC O157:H7. The authors initially attached fluorescent imager and compact laser-diode-based photosource into a smartphone. Included in the assembly were excitation light resource, which illuminated the cuvette, and a signal collection system. FITC-labeled rabbit polyclonal Ab and monoclonal Ab-magnetic beads were allowed to bind to *E. coli* O157:H7 forming a sandwich-type complex. Fluorescent signals generated from the samples were collected and recorded by the built-in lens and sensor chip. The picture processing program analyzed the average fluorescence intensity which achieved a LOD of 10 CFU/ml or g when applied on milk and egg samples. The technique may experience cross-reactivity, as commonly reported from using an immune-based approach and the instability of antibodies may also affect the performance of the technology if routinely used for screening target pathogens.

**Figure 6:**
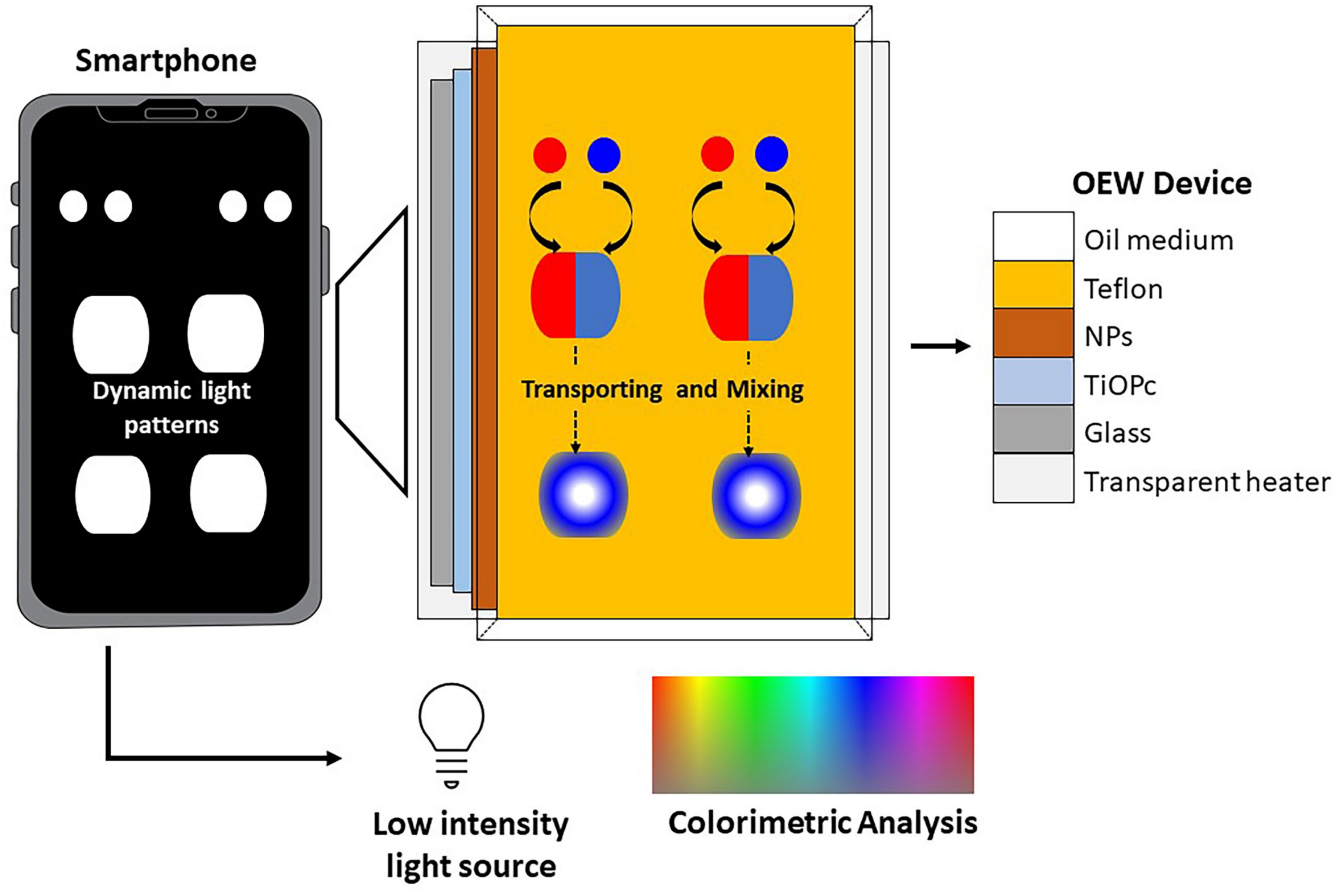
A low-cost lab-on-a-smartphone (LOS) for on-site monitoring of *E. coli* in environmental water, which was comprised of plasmonic-enhanced optoelectrowetting (OEW) device (Thio et al., 2022).

A low-cost lab-on-a-smartphone (LOS) for on-site monitoring of *E. coli* in environmental water, which was comprised of plasmonic-enhanced optoelectrowetting (OEW) device, a transparent heater (65^o^ C), and a smartphone was reported by Thio et al. (2022). The water monitoring system in the compact device was facilitated by LAMP assays. The OEW provided a tubeless and pumpless operation for sample preparation. The smartphone acted as an optical detector during LAMP assays *via* digital images and simultaneously conducted a red-green-blue analysis for a quantitative colorimetric study using its image processing app. Positive samples containing amplified *E. coli* DNA exhibited color change that was visible in the naked eye (Figure 6).

The clustered regularly interspaced short palindromic repeats (CRISPR)/Cas system has been recently combined with nucleic acid-based biosensors in food safety (Mao et al., 2022). This technology can accurately identify the presence of target nucleic acids and sequence variations, which is significant in biosensing applications. Lee and Oh (2022) developed a fluorescence-based detection technology that utilized a combination of filtration, DNA extraction, and LAMP-CRISPR/Cas12a system to screen for the presence of *E. coli* O157:H7 in romaine lettuce. The technology achieved a LOD of 4.80 × 10 CFU/g and required 70 min testing time. To improve the sensitivity of magnetic relaxation switching (MRS) biosensors, Shen et al. (2022) employed CRISPR-Cas12a system to precisely control the binding of magnetic nanoparticles (MNPs) needed for detecting *S.* Typhimurium in chicken meat samples. The CRISPR-MRS biosensor allowed the detection of *S.* Typhimurium by MRS measurement as well as a visual evaluation based on the dispersion state of MNPs with a LOD of 1.3 × 10^2^ CFU/ml. Though the approach included cumbersome steps, a future investigation may simplify the process to provide flexibility, especially for handling a large volume of samples.

Artificial intelligence (AI) classification algorithms-assisted hyperspectral microscopic imaging (HMI) was developed by Kang et al. (2021) to rapidly identify foodborne pathogens (*C. jejuni*, *E. coli*, *L. innocua*, *S.* Typhimurium, and *S. aureus*). The self-assembled HMI system was utilized for hypercube image scanning of the pathogens. The regions of interest (ROI) included whole-cell ROI, boundary ROI (outer membrane of cells), and center ROI (inner area of cells). These ROIs were investigated to evaluate classification performance. The long-short term memory (LSTM) network was named by an artificial recurrent neural network that processed the data from various ROIs. The AI-based classifier was able to achieve its highest accuracy of 92.9% for the center ROI dataset, which can also instantly predict spectra, hence efficiently and immediately predicting and identifying live foodborne pathogens at the single-cell level. Similarly, Raman spectroscopy and machine learning algorithm was used by Sun et al. (2023) to identify *Salmonella* spp. rapidly. Raman spectroscopy was applied to acquire spectral data and chose convolutional neural network (CNN) to solve multi-classification problems and conduct in-depth mining and analysis. The effects of five spectral preprocessing methods were compared, wherein Savitzky–Golay (SG) smoothing combined with Standard Normal Variate (SNV) was chosen as the best predictor of *Salmonella* serotypes.

SG combined with SNV achieved an accuracy of 98.7% for the training set and over 98.5% for the test set in CNN model.

## Summary and outlook

Portable biosensors offer rapid screening and measurement capabilities of foodborne pathogens and other biological hazards on various matrices, within minutes or hours, with or without pre-treatment steps. Due to the handheld and portability features, biosensors can be applied in the field without the need to process samples in the laboratory. The key features of portable biosensors such as miniaturized, handheld, and accessible tools, allow remote and wireless networking capabilities which are essential in handling a high volume of food samples from multiple locations. Microfluidic, nanotechnology, and novel biological design approaches, including smartphones and Apps, have introduced compatibility and ease of use to the newly developed portable biosensors for end users. The “laboratory-free” and “decentralized” concepts can process samples with simple steps and micro volumes at a fraction of the cost of traditional methods. The market size of global food safety testing steadily increases, and the continuous occurrence of outbreaks related to foodborne illnesses has remained its main driving force. New materials for biorecognition purposes are being discovered and explored at a faster rate. These new options are continuously being improved in terms of performance while keeping a competitive production cost. Many of these newly developed and advanced biosensors will encounter hurdles as it enters the market, and some may not be able to reach their commercialization stage. Thus, it is critical that developers and researchers adapt to the ever-growing and changing needs of the industry. Last, these mentioned innovations are on the right track to keep improving the current and available detection and screening technologies specifically for outbreak-causing foodborne pathogens and reducing the highly-preventable infection from consuming contaminated food products.

## Author contributions

IQ conducted the literature review and wrote the manuscript. TV prepared the figures. IQ, TV, C-SL, and VW revised the manuscript, added important scientific content, and refined the interpretation of the results, led in conceptualization, funding acquisition, review and editing of the manuscript, supervision, and project administration. All authors contributed to the article and approved the submitted version.

## Funding

This work was supported by the United States Department of Agriculture (USDA NIFA AFRI) food safety grant (award number 2015-69003-32075).

## Conflict of interest

The authors declare that the research was conducted in the absence of any commercial or financial relationships that could be construed as a potential conflict of interest.

## Publisher’s note

All claims expressed in this article are solely those of the authors and do not necessarily represent those of their affiliated organizations, or those of the publisher, the editors and the reviewers. Any product that may be evaluated in this article, or claim that may be made by its manufacturer, is not guaranteed or endorsed by the publisher.
